# On a Simple Means of Arresting Bleeding from Leech Bites

**Published:** 1845-12

**Authors:** 


*On a Simple Means of arresting Bleeding from Leech Bites.* The troublesome and occasionally fatal effects of hemorrhage from leech bites, especially in children, has led to a great number of devices for the purpose of arresting the bleeding. Mr. Gosset recommends the following plan, which from its extreme simplicity, is worthy of universal publicity. "After wiping away the blood he applies quickly a piece of visiting card, cut into a circular form about the size of a silver penny, the glazed side being applied to the wound. This must be pressed firmly into the wound, and held there about a minute; it will then become glued to the part and effectually restrain further hemorrhage." In this way Mr. Gosset has continually arrested hemorrhages which had resisted caustic, acetic acid, &c.—*Lancet.*—*Ranking's Abstract.*

